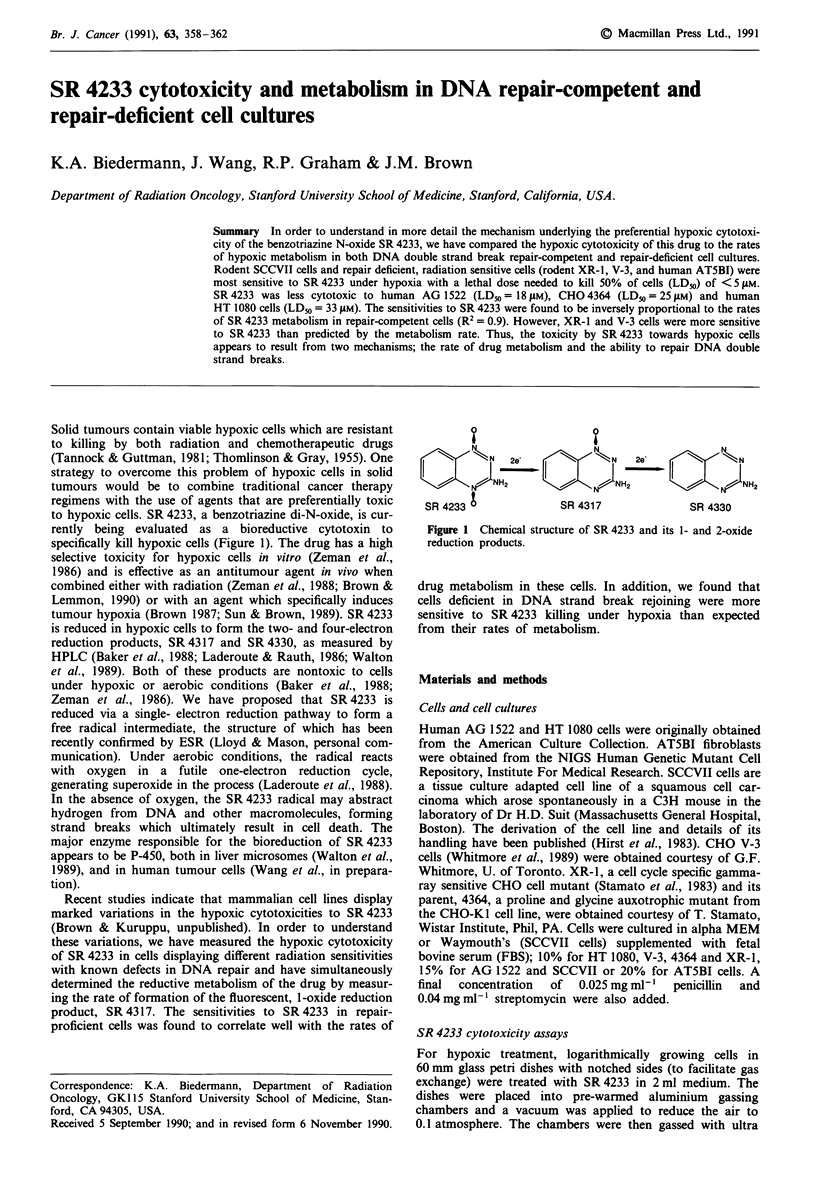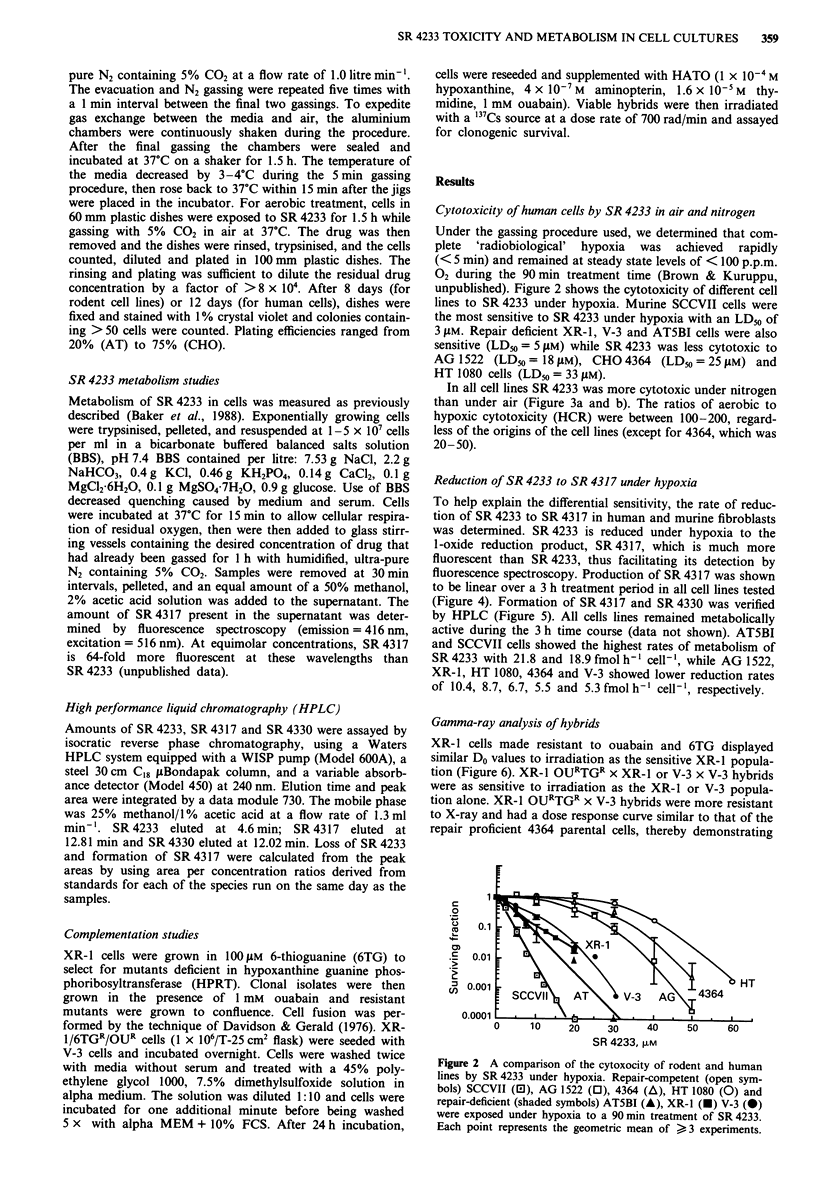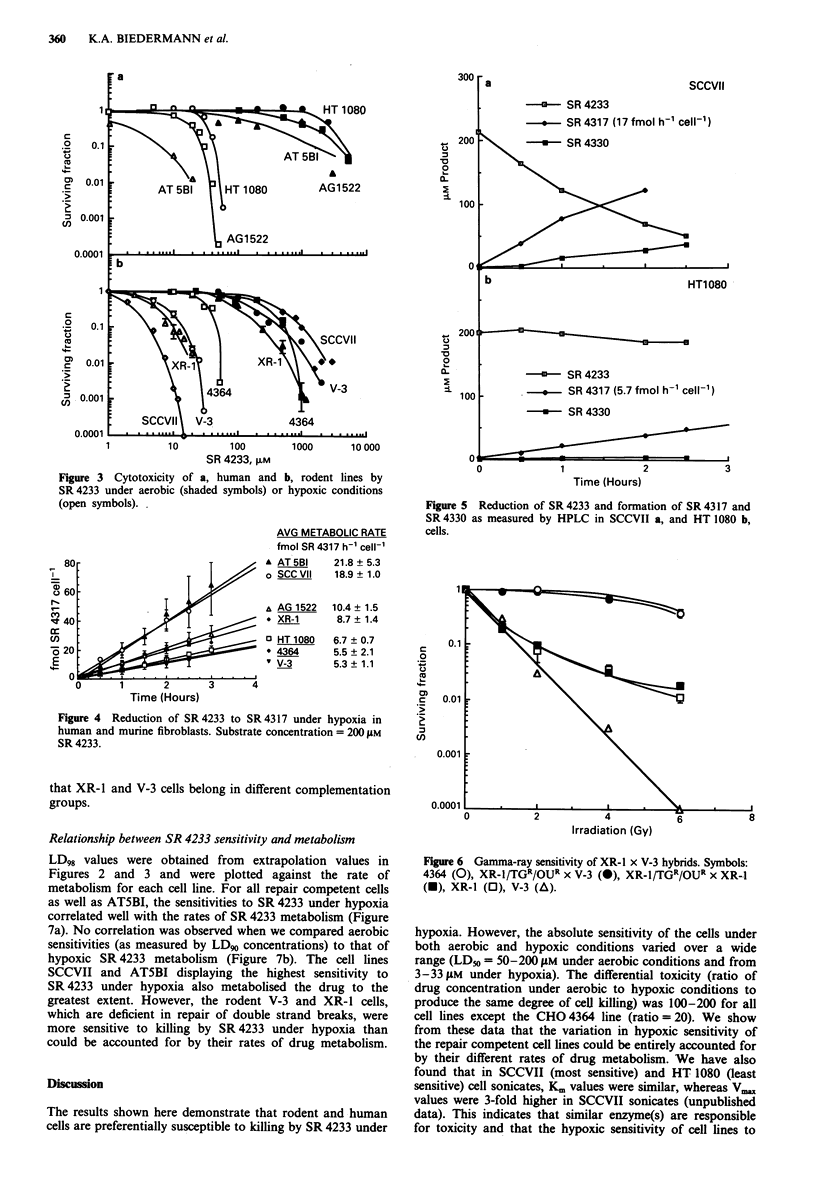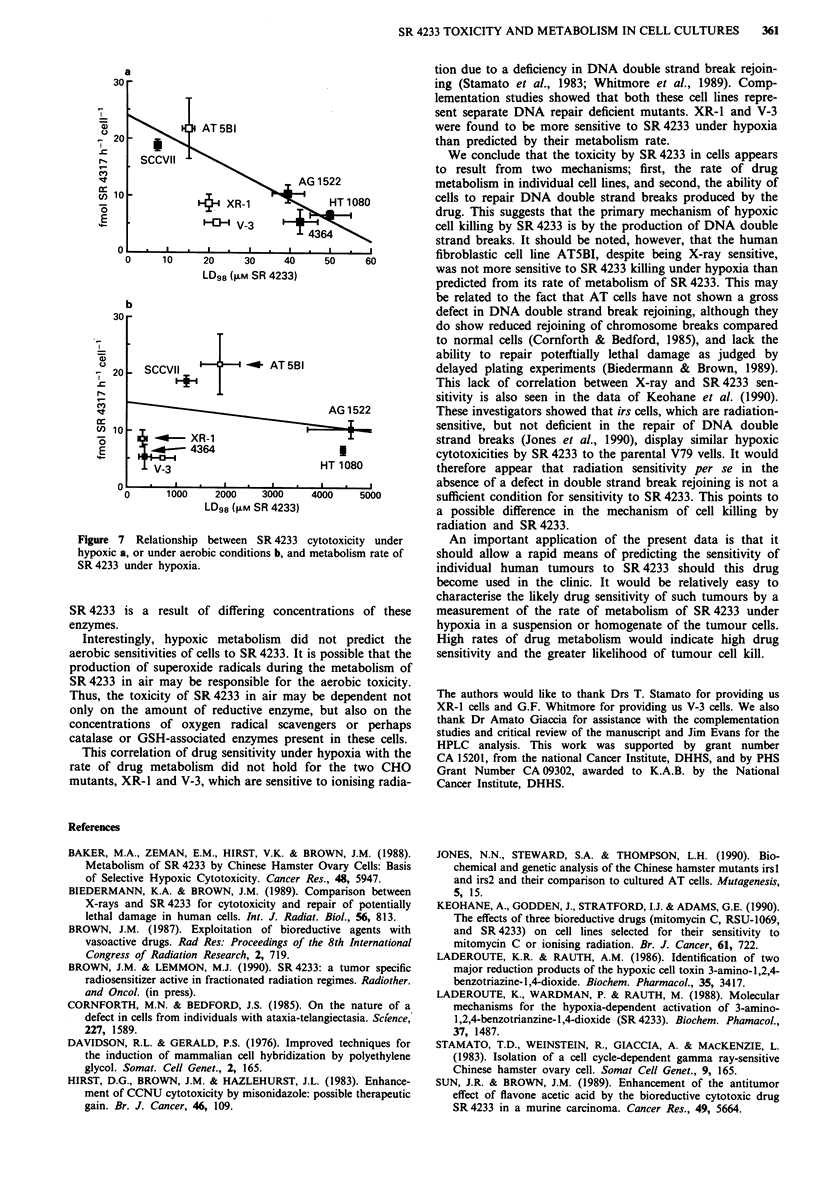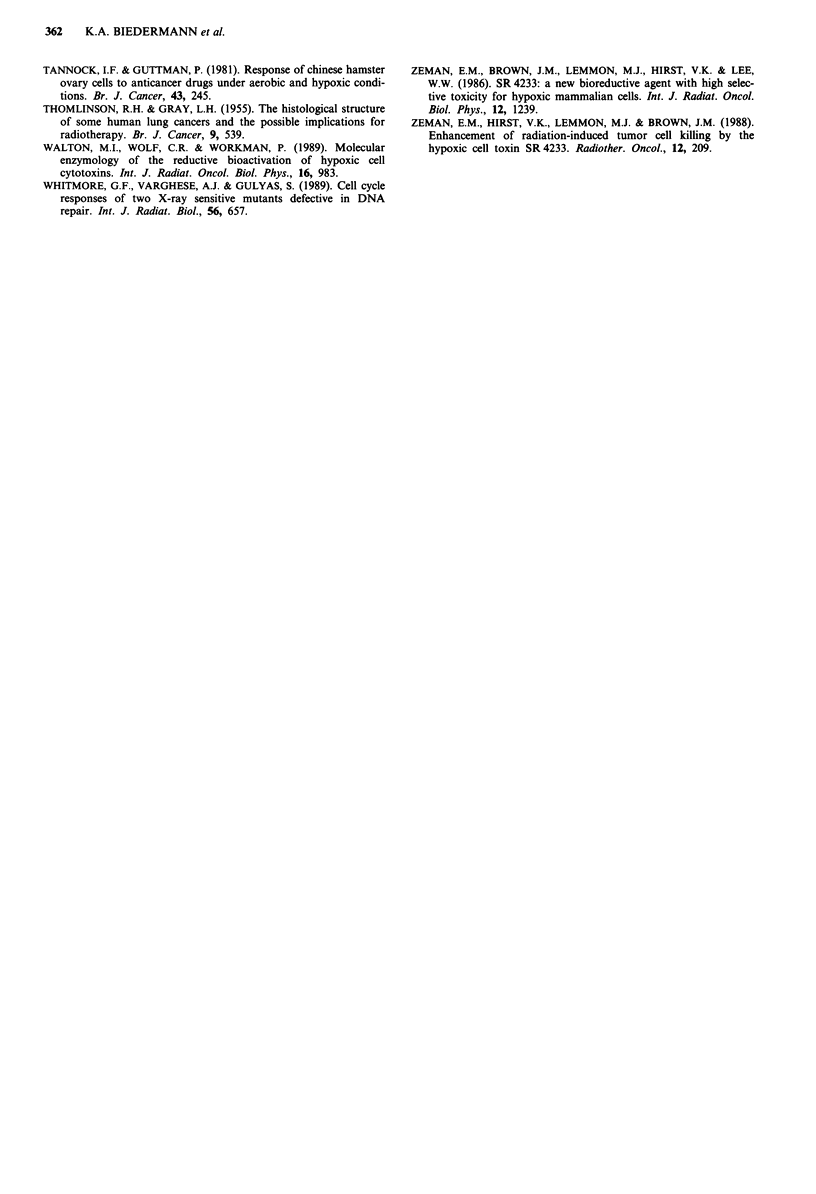# SR 4233 cytotoxicity and metabolism in DNA repair-competent and repair-deficient cell cultures.

**DOI:** 10.1038/bjc.1991.85

**Published:** 1991-03

**Authors:** K. A. Biedermann, J. Wang, R. P. Graham, J. M. Brown

**Affiliations:** Department of Radiation Oncology, Stanford University School of Medicine, California 94305.

## Abstract

In order to understand in more detail the mechanism underlying the preferential hypoxic cytotoxicity of the benzotriazine N-oxide SR 4233, we have compared the hypoxic cytotoxicity of this drug to the rates of hypoxic metabolism in both DNA double strand break repair-competent and repair-deficient cell cultures. Rodent SCCVII cells and repair deficient, radiation sensitive cells (rodent XR-1, V-3, and human AT5BI) were most sensitive to SR 4233 under hypoxia with a lethal dose needed to kill 50% of cells (LD50) of less than 5 microM. SR 4233 was less cytotoxic to human AG 1522 (LD50 = 18 microM), CHO 4364 (LD50 = 25 microM) and human HT 1080 cells (LD50 = 33 microM). The sensitivities to SR 4233 were found to be inversely proportional to the rates of SR 4233 metabolism in repair-competent cells (R2 = 0.9). However, XR-1 and V-3 cells were more sensitive to SR 4233 than predicted by the metabolism rate. Thus, the toxicity by SR 4233 towards hypoxic cells appears to result from two mechanisms; the rate of drug metabolism and the ability to repair DNA double strand breaks.


Br. J. Cancer (1991), 63, 358-362 © Macmillan Press Ltd., 1991

### SR 4233 cytotoxicity and metabolism in DNA repair-competent and repair-deficient cell cultures

K.A. Biedermann, J. Wang, R.P. Graham & J.M. Brown

*Department of Radiation Oncology, Stanford University School of Medicine, Stanford, California, USA.*

**Summary** In order to understand in more detail the mechanism underlying the preferential hypoxic cytotoxicity of the benzotriazine N-oxide SR 4233, we have compared the hypoxic cytotoxicity of this drug to the rates of hypoxic metabolism in both DNA double strand break repair-competent and repair-deficient cell cultures. Rodent SCCVII cells and repair deficient, radiation sensitive cells (rodent XR-1, V-3, and human AT5BI) were most sensitive to SR 4233 under hypoxia with a lethal dose needed to kill 50% of cells ($LD_{50}$) of $< 5\ \mu M$. SR 4233 was less cytotoxic to human AG 1522 ($LD_{50} = 18\ \mu M$), CHO 4364 ($LD_{50} = 25\ \mu M$) and human HT 1080 cells ($LD_{50} = 33\ \mu M$). The sensitivities to SR 4233 were found to be inversely proportional to the rates of SR 4233 metabolism in repair-competent cells ($R^2 = 0.9$). However, XR-1 and V-3 cells were more sensitive to SR 4233 than predicted by the metabolism rate. Thus, the toxicity by SR 4233 towards hypoxic cells appears to result from two mechanisms; the rate of drug metabolism and the ability to repair DNA double strand breaks.

Solid tumours contain viable hypoxic cells which are resistant to killing by both radiation and chemotherapeutic drugs (Tannock & Guttman, 1981; Thomlinson & Gray, 1955). One strategy to overcome this problem of hypoxic cells in solid tumours would be to combine traditional cancer therapy regimens with the use of agents that are preferentially toxic to hypoxic cells. SR 4233, a benzotriazine di-N-oxide, is currently being evaluated as a bioreductive cytotoxin to specifically kill hypoxic cells (Figure 1). The drug has a high selective toxicity for hypoxic cells *in vitro* (Zeman *et al.*, 1986) and is effective as an antitumour agent *in vivo* when combined either with radiation (Zeman *et al.*, 1988; Brown & Lemmon, 1990) or with an agent which specifically induces tumour hypoxia (Brown 1987; Sun & Brown, 1989). SR 4233 is reduced in hypoxic cells to form the two- and four-electron reduction products, SR 4317 and SR 4330, as measured by HPLC (Baker *et al.*, 1988; Laderoute & Rauth, 1986; Walton *et al.*, 1989). Both of these products are nontoxic to cells under hypoxic or aerobic conditions (Baker *et al.*, 1988; Zeman *et al.*, 1986). We have proposed that SR 4233 is reduced via a single- electron reduction pathway to form a free radical intermediate, the structure of which has been recently confirmed by ESR (Lloyd & Mason, personal communication). Under aerobic conditions, the radical reacts with oxygen in a futile one-electron reduction cycle, generating superoxide in the process (Laderoute *et al.*, 1988). In the absence of oxygen, the SR 4233 radical may abstract hydrogen from DNA and other macromolecules, forming strand breaks which ultimately result in cell death. The major enzyme responsible for the bioreduction of SR 4233 appears to be P-450, both in liver microsomes (Walton *et al.*, 1989), and in human tumour cells (Wang *et al.*, in preparation).

Recent studies indicate that mammalian cell lines display marked variations in the hypoxic cytotoxicities to SR 4233 (Brown & Kuruppu, unpublished). In order to understand these variations, we have measured the hypoxic cytotoxicity of SR 4233 in cells displaying different radiation sensitivities with known defects in DNA repair and have simultaneously determined the reductive metabolism of the drug by measuring the rate of formation of the fluorescent, 1-oxide reduction product, SR 4317. The sensitivities to SR 4233 in repair-proficient cells was found to correlate well with the rates of drug metabolism in these cells. In addition, we found that cells deficient in DNA strand break rejoining were more sensitive to SR 4233 killing under hypoxia than expected from their rates of metabolism.

**Figure 1** Chemical structure of SR 4233 and its 1- and 2-oxide reduction products.

### Materials and methods

### *Cells and cell cultures*

Human AG 1522 and HT 1080 cells were originally obtained from the American Culture Collection. AT5BI fibroblasts were obtained from the NIGS Human Genetic Mutant Cell Repository, Institute For Medical Research. SCCVII cells are a tissue culture adapted cell line of a squamous cell carcinoma which arose spontaneously in a C3H mouse in the laboratory of Dr H.D. Suit (Massachusetts General Hospital, Boston). The derivation of the cell line and details of its handling have been published (Hirst *et al.*, 1983). CHO V-3 cells (Whitmore *et al.*, 1989) were obtained courtesy of G.F. Whitmore, U. of Toronto. XR-1, a cell cycle specific gamma-ray sensitive CHO cell mutant (Stamato *et al.*, 1983) and its parent, 4364, a proline and glycine auxotrophic mutant from the CHO-K1 cell line, were obtained courtesy of T. Stamato, Wistar Institute, Phil, PA. Cells were cultured in alpha MEM or Waymouth's (SCCVII cells) supplemented with fetal bovine serum (FBS); 10% for HT 1080, V-3, 4364 and XR-1, 15% for AG 1522 and SCCVII or 20% for AT5BI cells. A final concentration of $0.025\ mg\ ml^{-1}$ penicillin and $0.04\ mg\ ml^{-1}$ streptomycin were also added.

### *SR 4233 cytotoxicity assays*

For hypoxic treatment, logarithmically growing cells in 60 mm glass petri dishes with notched sides (to facilitate gas exchange) were treated with SR 4233 in 2 ml medium. The dishes were placed into pre-warmed aluminium gassing chambers and a vacuum was applied to reduce the air to 0.1 atmosphere. The chambers were then gassed with ultra

Correspondence: K.A. Biedermann, Department of Radiation Oncology, GK115 Stanford University School of Medicine, Stanford, CA 94305, USA.
Received 5 September 1990; and in revised form 6 November 1990.

pure $N_2$ containing 5% $CO_2$ at a flow rate of 1.0 litre $min^{-1}$. The evacuation and $N_2$ gassing were repeated five times with a 1 min interval between the final two gassings. To expedite gas exchange between the media and air, the aluminium chambers were continuously shaken during the procedure. After the final gassing the chambers were sealed and incubated at 37°C on a shaker for 1.5 h. The temperature of the media decreased by 3–4°C during the 5 min gassing procedure, then rose back to 37°C within 15 min after the jigs were placed in the incubator. For aerobic treatment, cells in 60 mm plastic dishes were exposed to SR 4233 for 1.5 h while gassing with 5% $CO_2$ in air at 37°C. The drug was then removed and the dishes were rinsed, trypsinised, and the cells counted, diluted and plated in 100 mm plastic dishes. The rinsing and plating was sufficient to dilute the residual drug concentration by a factor of $>8 \times 10^4$. After 8 days (for rodent cell lines) or 12 days (for human cells), dishes were fixed and stained with 1% crystal violet and colonies containing $>50$ cells were counted. Plating efficiencies ranged from 20% (AT) to 75% (CHO).

### *SR 4233 metabolism studies*

Metabolism of SR 4233 in cells was measured as previously described (Baker *et al.*, 1988). Exponentially growing cells were trypsinised, pelleted, and resuspended at $1-5 \times 10^7$ cells per ml in a bicarbonate buffered balanced salts solution (BBS), pH 7.4 BBS contained per litre: 7.53 g NaCl, 2.2 g $NaHCO_3$, 0.4 g KCl, 0.46 g $KH_2PO_4$, 0.14 g $CaCl_2$, 0.1 g $MgCl_2 \cdot 6H_2O$, 0.1 g $MgSO_4 \cdot 7H_2O$, 0.9 g glucose. Use of BBS decreased quenching caused by medium and serum. Cells were incubated at 37°C for 15 min to allow cellular respiration of residual oxygen, then were then added to glass stirring vessels containing the desired concentration of drug that had already been gassed for 1 h with humidified, ultra-pure $N_2$ containing 5% $CO_2$. Samples were removed at 30 min intervals, pelleted, and an equal amount of a 50% methanol, 2% acetic acid solution was added to the supernatant. The amount of SR 4317 present in the supernatant was determined by fluorescence spectroscopy (emission = 416 nm, excitation = 516 nm). At equimolar concentrations, SR 4317 is 64-fold more fluorescent at these wavelengths than SR 4233 (unpublished data).

### *High performance liquid chromatography (HPLC)*

Amounts of SR 4233, SR 4317 and SR 4330 were assayed by isocratic reverse phase chromatography, using a Waters HPLC system equipped with a WISP pump (Model 600A), a steel 30 cm $C_{18}$ μBondapak column, and a variable absorbance detector (Model 450) at 240 nm. Elution time and peak area were integrated by a data module 730. The mobile phase was 25% methanol/1% acetic acid at a flow rate of 1.3 ml $min^{-1}$. SR 4233 eluted at 4.6 min; SR 4317 eluted at 12.81 min and SR 4330 eluted at 12.02 min. Loss of SR 4233 and formation of SR 4317 were calculated from the peak areas by using area per concentration ratios derived from standards for each of the species run on the same day as the samples.

### *Complementation studies*

XR-1 cells were grown in 100 μM 6-thioguanine (6TG) to select for mutants deficient in hypoxanthine guanine phosphoribosyltransferase (HPRT). Clonal isolates were then grown in the presence of 1 mM ouabain and resistant mutants were grown to confluence. Cell fusion was performed by the technique of Davidson & Gerald (1976). XR-1/6TG$^R$/OU$^R$ cells ($1 \times 10^6$/T-25 $cm^2$ flask) were seeded with V-3 cells and incubated overnight. Cells were washed twice with media without serum and treated with a 45% polyethylene glycol 1000, 7.5% dimethylsulfoxide solution in alpha medium. The solution was diluted 1:10 and cells were incubated for one additional minute before being washed 5 × with alpha MEM + 10% FCS. After 24 h incubation, cells were reseeded and supplemented with HATO ($1 \times 10^{-4}$ M hypoxanthine, $4 \times 10^{-7}$ M aminopterin, $1.6 \times 10^{-5}$ M thymidine, 1 mM ouabain). Viable hybrids were then irradiated with a $^{137}Cs$ source at a dose rate of 700 rad/min and assayed for clonogenic survival.

### Results

### *Cytotoxicity of human cells by SR 4233 in air and nitrogen*

Under the gassing procedure used, we determined that complete 'radiobiological' hypoxia was achieved rapidly (<5 min) and remained at steady state levels of <100 p.p.m. $O_2$ during the 90 min treatment time (Brown & Kuruppu, unpublished). Figure 2 shows the cytotoxicity of different cell lines to SR 4233 under hypoxia. Murine SCCVII cells were the most sensitive to SR 4233 under hypoxia with an $LD_{50}$ of 3 μM. Repair deficient XR-1, V-3 and AT5BI cells were also sensitive ($LD_{50}$ = 5 μM) while SR 4233 was less cytotoxic to AG 1522 ($LD_{50}$ = 18 μM), CHO 4364 ($LD_{50}$ = 25 μM) and HT 1080 cells ($LD_{50}$ = 33 μM).

In all cell lines SR 4233 was more cytotoxic under nitrogen than under air (Figure 3a and b). The ratios of aerobic to hypoxic cytotoxicity (HCR) were between 100–200, regardless of the origins of the cell lines (except for 4364, which was 20–50).

### *Reduction of SR 4233 to SR 4317 under hypoxia*

To help explain the differential sensitivity, the rate of reduction of SR 4233 to SR 4317 in human and murine fibroblasts was determined. SR 4233 is reduced under hypoxia to the 1-oxide reduction product, SR 4317, which is much more fluorescent than SR 4233, thus facilitating its detection by fluorescence spectroscopy. Production of SR 4317 was shown to be linear over a 3 h treatment period in all cell lines tested (Figure 4). Formation of SR 4317 and SR 4330 was verified by HPLC (Figure 5). All cells lines remained metabolically active during the 3 h time course (data not shown). AT5BI and SCCVII cells showed the highest rates of metabolism of SR 4233 with 21.8 and 18.9 fmol $h^{-1}$ $cell^{-1}$, while AG 1522, XR-1, HT 1080, 4364 and V-3 showed lower reduction rates of 10.4, 8.7, 6.7, 5.5 and 5.3 fmol $h^{-1}$ $cell^{-1}$, respectively.

### *Gamma-ray analysis of hybrids*

XR-1 cells made resistant to ouabain and 6TG displayed similar $D_0$ values to irradiation as the sensitive XR-1 population (Figure 6). XR-1 OU$^R$TG$^R$ × XR-1 or V-3 × V-3 hybrids were as sensitive to irradiation as the XR-1 or V-3 population alone. XR-1 OU$^R$TG$^R$ × V-3 hybrids were more resistant to X-ray and had a dose response curve similar to that of the repair proficient 4364 parental cells, thereby demonstrating

**Figure 2** A comparison of the cytoxocity of rodent and human lines by SR 4233 under hypoxia. Repair-competent (open symbols) SCCVII (◻), AG 1522 (□), 4364 (△), HT 1080 (○) and repair-deficient (shaded symbols) AT5BI (▲), XR-1 (■) V-3 (●) were exposed under hypoxia to a 90 min treatment of SR 4233. Each point represents the geometric mean of ⩾3 experiments.

**Figure 3** Cytotoxicity of **a**, human and **b**, rodent lines by SR 4233 under aerobic (shaded symbols) or hypoxic conditions (open symbols).

**Figure 4** Reduction of SR 4233 to SR 4317 under hypoxia in human and murine fibroblasts. Substrate concentration = 200 μM SR 4233.

**Figure 5** Reduction of SR 4233 and formation of SR 4317 and SR 4330 as measured by HPLC in SCCVII **a**, and HT 1080 **b**, cells.

**Figure 6** Gamma-ray sensitivity of XR-1 × V-3 hybrids. Symbols: 4364 (○), XR-1/TG$^R$/OU$^R$ × V-3 (●), XR-1/TG$^R$/OU$^R$ × XR-1 (■), XR-1 (□), V-3 (△).

that XR-1 and V-3 cells belong in different complementation groups.

*Relationship between SR 4233 sensitivity and metabolism*

$LD_{98}$ values were obtained from extrapolation values in Figures 2 and 3 and were plotted against the rate of metabolism for each cell line. For all repair competent cells as well as AT5BI, the sensitivities to SR 4233 under hypoxia correlated well with the rates of SR 4233 metabolism (Figure 7a). No correlation was observed when we compared aerobic sensitivities (as measured by $LD_{90}$ concentrations) to that of hypoxic SR 4233 metabolism (Figure 7b). The cell lines SCCVII and AT5BI displaying the highest sensitivity to SR 4233 under hypoxia also metabolised the drug to the greatest extent. However, the rodent V-3 and XR-1 cells, which are deficient in repair of double strand breaks, were more sensitive to killing by SR 4233 under hypoxia than could be accounted for by their rates of drug metabolism.

### Discussion

The results shown here demonstrate that rodent and human cells are preferentially susceptible to killing by SR 4233 under hypoxia. However, the absolute sensitivity of the cells under both aerobic and hypoxic conditions varied over a wide range ($LD_{50}$ = 50–200 μM under aerobic conditions and from 3–33 μM under hypoxia). The differential toxicity (ratio of drug concentration under aerobic to hypoxic conditions to produce the same degree of cell killing) was 100–200 for all cell lines except the CHO 4364 line (ratio = 20). We show from these data that the variation in hypoxic sensitivity of the repair competent cell lines could be entirely accounted for by their different rates of drug metabolism. We have also found that in SCCVII (most sensitive) and HT 1080 (least sensitive) cell sonicates, $K_m$ values were similar, whereas $V_{max}$ values were 3-fold higher in SCCVII sonicates (unpublished data). This indicates that similar enzyme(s) are responsible for toxicity and that the hypoxic sensitivity of cell lines to

**Figure 7** Relationship between SR 4233 cytotoxicity under hypoxic **a**, or under aerobic conditions **b**, and metabolism rate of SR 4233 under hypoxia.

SR 4233 is a result of differing concentrations of these enzymes.

Interestingly, hypoxic metabolism did not predict the aerobic sensitivities of cells to SR 4233. It is possible that the production of superoxide radicals during the metabolism of SR 4233 in air may be responsible for the aerobic toxicity. Thus, the toxicity of SR 4233 in air may be dependent not only on the amount of reductive enzyme, but also on the concentrations of oxygen radical scavengers or perhaps catalase or GSH-associated enzymes present in these cells.

This correlation of drug sensitivity under hypoxia with the rate of drug metabolism did not hold for the two CHO mutants, XR-1 and V-3, which are sensitive to ionising radiation due to a deficiency in DNA double strand break rejoining (Stamato *et al.*, 1983; Whitmore *et al.*, 1989). Complementation studies showed that both these cell lines represent separate DNA repair deficient mutants. XR-1 and V-3 were found to be more sensitive to SR 4233 under hypoxia than predicted by their metabolism rate.

We conclude that the toxicity by SR 4233 in cells appears to result from two mechanisms; first, the rate of drug metabolism in individual cell lines, and second, the ability of cells to repair DNA double strand breaks produced by the drug. This suggests that the primary mechanism of hypoxic cell killing by SR 4233 is by the production of DNA double strand breaks. It should be noted, however, that the human fibroblastic cell line AT5BI, despite being X-ray sensitive, was not more sensitive to SR 4233 killing under hypoxia than predicted from its rate of metabolism of SR 4233. This may be related to the fact that AT cells have not shown a gross defect in DNA double strand break rejoining, although they do show reduced rejoining of chromosome breaks compared to normal cells (Cornforth & Bedford, 1985), and lack the ability to repair potentially lethal damage as judged by delayed plating experiments (Biedermann & Brown, 1989). This lack of correlation between X-ray and SR 4233 sensitivity is also seen in the data of Keohane *et al.* (1990). These investigators showed that *irs* cells, which are radiation-sensitive, but not deficient in the repair of DNA double strand breaks (Jones *et al.*, 1990), display similar hypoxic cytotoxicities by SR 4233 to the parental V79 vells. It would therefore appear that radiation sensitivity *per se* in the absence of a defect in double strand break rejoining is not a sufficient condition for sensitivity to SR 4233. This points to a possible difference in the mechanism of cell killing by radiation and SR 4233.

An important application of the present data is that it should allow a rapid means of predicting the sensitivity of individual human tumours to SR 4233 should this drug become used in the clinic. It would be relatively easy to characterise the likely drug sensitivity of such tumours by a measurement of the rate of metabolism of SR 4233 under hypoxia in a suspension or homogenate of the tumour cells. High rates of drug metabolism would indicate high drug sensitivity and the greater likelihood of tumour cell kill.

The authors would like to thank Drs T. Stamato for providing us XR-1 cells and G.F. Whitmore for providing us V-3 cells. We also thank Dr Amato Giaccia for assistance with the complementation studies and critical review of the manuscript and Jim Evans for the HPLC analysis. This work was supported by grant number CA 15201, from the national Cancer Institute, DHHS, and by PHS Grant Number CA 09302, awarded to K.A.B. by the National Cancer Institute, DHHS.

### References

BAKER, M.A., ZEMAN, E.M., HIRST, V.K. & BROWN, J.M. (1988). Metabolism of SR 4233 by Chinese Hamster Ovary Cells: Basis of Selective Hypoxic Cytotoxicity. *Cancer Res.*, **48**, 5947.

BIEDERMANN, K.A. & BROWN, J.M. (1989). Comparison between X-rays and SR 4233 for cytotoxicity and repair of potentially lethal damage in human cells. *Int. J. Radiat. Biol.*, **56**, 813.

BROWN, J.M. (1987). Exploitation of bioreductive agents with vasoactive drugs. *Rad Res: Proceedings of the 8th International Congress of Radiation Research*, **2**, 719.

BROWN, J.M. & LEMMON, M.J. (1990). SR 4233: a tumor specific radiosensitizer active in fractionated radiation regimes. *Radiother. and Oncol.* (in press).

CORNFORTH, M.N. & BEDFORD, J.S. (1985). On the nature of a defect in cells from individuals with ataxia-telangiectasia. *Science*, **227**, 1589.

DAVIDSON, R.L. & GERALD, P.S. (1976). Improved techniques for the induction of mammalian cell hybridization by polyethylene glycol. *Somat. Cell Genet.*, **2**, 165.

HIRST, D.G., BROWN, J.M. & HAZLEHURST, J.L. (1983). Enhancement of CCNU cytotoxicity by misonidazole: possible therapeutic gain. *Br. J. Cancer*, **46**, 109.

JONES, N.N., STEWARD, S.A. & THOMPSON, L.H. (1990). Biochemical and genetic analysis of the Chinese hamster mutants irs1 and irs2 and their comparison to cultured AT cells. *Mutagenesis*, **5**, 15.

KEOHANE, A., GODDEN, J., STRATFORD, I.J. & ADAMS, G.E. (1990). The effects of three bioreductive drugs (mitomycin C, RSU-1069, and SR 4233) on cell lines selected for their sensitivity to mitomycin C or ionising radiation. *Br. J. Cancer*, **61**, 722.

LADEROUTE, K.R. & RAUTH, A.M. (1986). Identification of two major reduction products of the hypoxic cell toxin 3-amino-1,2,4-benzotriazine-1,4-dioxide. *Biochem. Pharmacol.*, **35**, 3417.

LADEROUTE, K., WARDMAN, P. & RAUTH, M. (1988). Molecular mechanisms for the hypoxia-dependent activation of 3-amino-1,2,4-benzotrianzine-1,4-dioxide (SR 4233). *Biochem. Phamacol.*, **37**, 1487.

STAMATO, T.D., WEINSTEIN, R., GIACCIA, A. & MACKENZIE, L. (1983). Isolation of a cell cycle-dependent gamma ray-sensitive Chinese hamster ovary cell. *Somat Cell Genet.*, **9**, 165.

SUN, J.R. & BROWN, J.M. (1989). Enhancement of the antitumor effect of flavone acetic acid by the bioreductive cytotoxic drug SR 4233 in a murine carcinoma. *Cancer Res.*, **49**, 5664.

TANNOCK, I.F. & GUTTMAN, P. (1981). Response of chinese hamster ovary cells to anticancer drugs under aerobic and hypoxic conditions. *Br. J. Cancer*, **43**, 245.

THOMLINSON, R.H. & GRAY, L.H. (1955). The histological structure of some human lung cancers and the possible implications for radiotherapy. *Br. J. Cancer*, **9**, 539.

WALTON, M.I., WOLF, C.R. & WORKMAN, P. (1989). Molecular enzymology of the reductive bioactivation of hypoxic cell cytotoxins. *Int. J. Radiat. Oncol. Biol. Phys.*, **16**, 983.

WHITMORE, G.F., VARGHESE, A.J. & GULYAS, S. (1989). Cell cycle responses of two X-ray sensitive mutants defective in DNA repair. *Int. J. Radiat. Biol.*, **56**, 657.

ZEMAN, E.M., BROWN, J.M., LEMMON, M.J., HIRST, V.K. & LEE, W.W. (1986). SR 4233: a new bioreductive agent with high selective toxicity for hypoxic mammalian cells. *Int. J. Radiat. Oncol. Biol. Phys.*, **12**, 1239.

ZEMAN, E.M., HIRST, V.K., LEMMON, M.J. & BROWN, J.M. (1988). Enhancement of radiation-induced tumor cell killing by the hypoxic cell toxin SR 4233. *Radiother. Oncol.*, **12**, 209.